# Clinical study on acupuncture treatment of gastrointestinal damp-heat acne

**DOI:** 10.1097/MD.0000000000027503

**Published:** 2021-11-05

**Authors:** Ping-Ping Duan, Chao-Qun Yan, Hui-Shang Feng, Yuan Chen, Ning Sun, Ya-Qi Yao, Kai-Bing Tian, Guang-An Wang

**Affiliations:** a School of Acupuncture-Moxibustion and Tuina, Henan University of Chinese Medicine, 63 Dongming Road, Jinshui District, Henan, China; b Department of Acupuncture and Moxibustion, Dongzhimen Hospital Beijing University of Chinese Medicine, Hai Yun Cang on the 5th Zip, Dongcheng District, Beijing, China; c Fourth Department of Obstetrics and Gynecology, Zhumadian Traditional Chinese Medicine Hospital, No.895 Jiefang Road, Yicheng District, Zhumadian, Henan Province, China; d Department of Neurosurgery, Capital Medical University, Beijing Tiantan Hospital, No. 199, Nan Si Huan Xi Road, Beijing 100010, China.

**Keywords:** acne, acupuncture, clinical trial, spleen and stomach guiyuan acupuncture method

## Abstract

**Background:**

Acne is a common inflammatory disease of sebaceous glands, which brings extensive emotional and psychological distress to patients. Although acupuncture has certain advantages in the treatment of acne, the curative effect is not exact. The purpose of this trial is to evaluate the feasibility, preliminary efficacy, and safety of the “Spleen and Stomach Guiyuan Acupuncture Method” (SSGA) in the treatment of gastrointestinal damp-heat acne.

**Methods:**

The proposed protocol is planned as a randomized, assessor-blind, conventional-treatment-controlled trial to evaluate the efficacy of SSGA on gastrointestinal damp-heat acne. Seventy six gastrointestinal damp-heat acne patients will be randomly divided into 2 groups and receive SSGA or conventional acupuncture treatment. The entire study period is 12 weeks, including an 8-week treatment period and a 4-week follow-up period. All patients will receive 16 sessions of acupuncture treatment over 8 weeks. The primary outcome is the investigation global assessment (IGA) at week 8, which is an overall assessment of the degree of the inflammatory and non-inflammatory lesion. The secondary outcomes include IGA, the total facial lesion count (Total Lesion Count), the acne-specific quality of life, etc at weeks 8 and 12. The Expectation and Credibility of treatment rating scale will be used to measure the patients’ attitudes to acupuncture after the first treatment. Adverse events will also be recorded.

**Discussion:**

This study is helpful to evaluate the feasibility, preliminary efficacy, and safety of SSGA in the treatment of gastrointestinal damp-heat acne. The results will be used in sample size calculations for subsequent large-scale studies.

Trial registration: Chinese Clinical Trial Registry ChiCTR2100047363. Registered on June 13, 2021.

## 1 Introduction

Acne is an inflammatory disease of sebaceous glands common in teenagers,^[1,2]^ mainly caused by hyperkeratosis of hair follicles, increased sebum secretion, and acne bacilli infection in hair follicles.^[3]^ It usually occurs on the face, chest, upper back, and neck, including mild, moderate, and severe. There are different inflammatory lesions at different stages, such as acne, blackheads, pustules, papules, and cystic nodules.^[4]^ Acne is the eighth most prevalent disease in the world, affecting 9.4% of the world's population.^[5]^ It can affect 85% of teenagers,^[1]^ of which moderate to severe accounts for about 20%.^[6]^ A Chinese survey revealed that the prevalence rate in the 19-year-old age group was as high as 46.8%.^[7]^ Though acne was mostly seen in adolescents, it was more common in women than men after the age of 23.^[8–11]^ As a common disease, it has a wide range of potential hazards and related costs. In mild cases, the symptoms are uncomfortable, affect the face, emotional and social psychological distress. In severe cases, they may have emotional and psychological distress, which even causes depression and suicide.^[5]^

At present, most of the treatments for acne are topical and oral retinoic acid, antibiotics and benzoyl peroxide, oral hormones or contraceptives, and so on.^[2]^ These medications generally require a long-term application. Sometimes, the side effects of these drugs are unavoidable, such as the possibility of drug resistance, dryness, peeling, erythema, and skin irritation. Furthermore, the recurrence rate is relatively high after treatment.^[10]^ With the development of medicine in recent years, some non-pharmacological therapies for acne are widely used, such as lasers, light devices including LED device, photodynamic therapy, intense pulsed light, and acupuncture.^[11]^ Acupuncture is widely used to treat various skin diseases.^[12]^ The World Health Organization has recommended acupuncture as an effective treatment for acne.^[13]^ Acupuncture, as a non-pharmacological therapy, has certain advantages in the treatment of acne and has fewer side effects. The existing randomized controlled trials have shown that acupuncture has a certain effect in the treatment of this disease.^[14,15]^ It can not only improve the skin damage of patients with acne but also improve the quality of life.^[16–20]^

However, the existing clinical studies have shown that the curative effect of acupuncture on acne is not exact. The effective rates of different acupuncture treatments are different. Some are relatively high, and some are relatively low. This is because these clinical studies did not adopt the principle of syndrome differentiation and treatment for the therapy of this disease, but only conventional treatment methods.^[16–20]^ The core of Traditional Chinese Medicine (TCM) is syndrome differentiation and treatment. According to the theory of TCM, acne is located in the skin, closely related to the lung, spleen, stomach, and intestine. The basic pathogenesis is heat toxin steaming the skin. The gastrointestinal damp-heat type is the most common in clinical practice. In line with the theory of TCM and clinical experience, the “Spleen and Stomach Guiyuan Acupuncture Method” (SSGA) selects acupoints according to dialectical classification and is mainly used to treat this syndrome. In addition, we found the SSGA is simple and easy to perform, and the effect is good in clinical practice. However, there is no relevant literature to report. Therefore, this trial intends to observe the clinical efficacy of gastrointestinal damp-heat acne through SSGA.

## 2 Materials and methods

### 2.1 Study design and ethics

**Figure 1:**
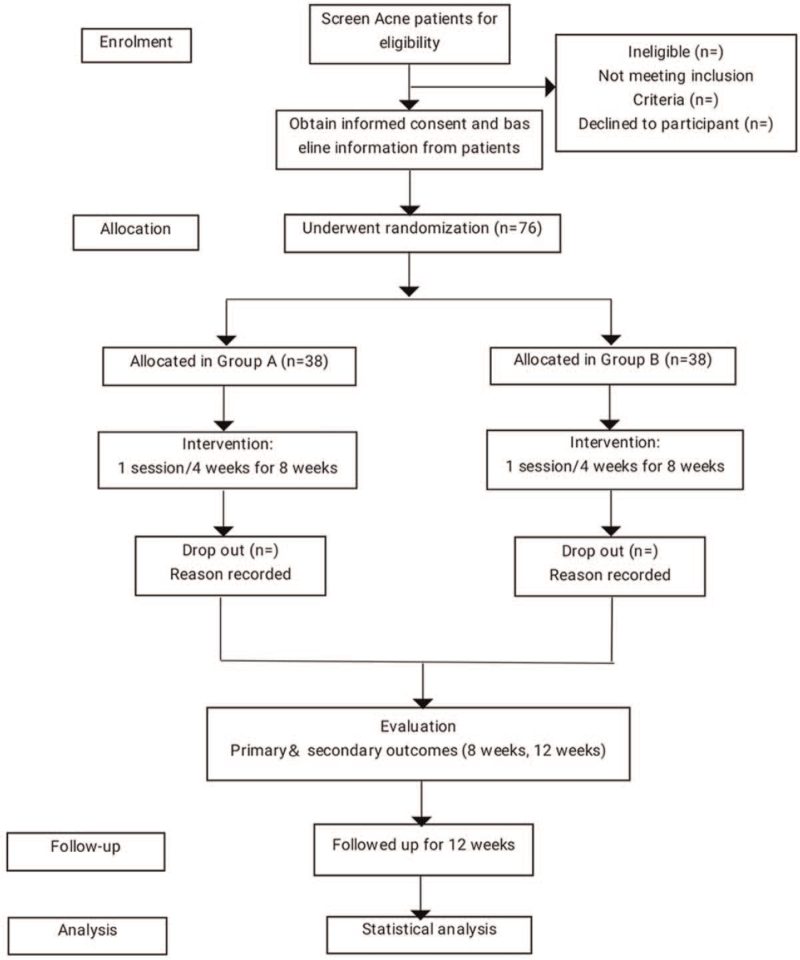
Flow chart.

The proposed study is a prospective, parallel, two-armed randomized controlled trial using a 1:1 allocation ratio with a 4-week follow-up. The study will be conducted at Dongzhimen Hospital of Beijing University of Chinese Medicine and the Third Affiliated Hospital of Henan University of Traditional Chinese Medicine. The outline of the study patient process is in Figure 1.

The research protocol was approved by the Medical Ethics Committee of Dongzhimen Hospital of Beijing University of Chinese Medicine (2021DZMEC-087-01) and by the Medical Ethics Committee Third Affiliated Hospital of Henan University of Chinese Medicine. We will obtain the patient's informed consent. Study results will be disseminated in peer-reviewed journals, through public events, and relevant third sector organizations.

### 2.2 Participants

#### 2.2.1 Western medicine diagnostic criteria

Patients with acne will refer to the Chinese Journal of Clinical Dermatology published in April 2017:

1. The age of onset is mostly male and female in adolescence.
2. The main affected parts are the face, front chest, and back of the neck.
3. The skin lesions are in the form of inflammatory papules, blackheads, whiteheads, pustules, cysts, or dark red nodules. In severe cases, there may even be scars, often accompanied by seborrhea, which is chronic.

#### 2.2.2 Diagnostic criteria for gastrointestinal damp-heat type in traditional Chinese medicine

With reference to the “Thirteenth Five-Year Plan” textbook “Traditional Chinese Medicine Surgery,” the diagnostic criteria for gastrointestinal damp-heat acne are as follows:

1. White or blackhead pimples on the face, chest, and back may be accompanied by light yellow or white lipid plugs; or red needle-like papules accompanied by redness and pain; or red papules accompanied by pustules.
2. With oily face; or with dry mouth and bad breath.
3. Thick stools, yellow urine.
4. The tongue is red, the coating is yellow and greasy, and the pulse is slippery.

#### 2.2.3 Inclusion criteria

Patients who meet the following conditions will be included:

1. 13 to 30 years old, male or female patients with acne.
2. Patients meet the diagnostic criteria for acne in Western medicine and the gastrointestinal damp-heat type according to the TCM syndrome differentiation criteria
3. Investigation Global Assessment (IGA) score is level 3 (moderate) or level 4 (severe).^[21]^
4. Symptoms have been on for more than 6 months.
5. Have a certain level of reading comprehension and be able to complete questionnaires independently.
6. Patients did not use acne drugs, such as oral, topical, or traditional Chinese medicine, within 4 weeks before entering the group.
7. Those who did not participate in other clinical studies within 1 month and were guaranteed not to participate in other clinical studies during this study period.

#### 2.2.4 Exclusion criteria

Patients who report the following conditions will be excluded:

1. Pregnant and lactating women, women who have the desire to give birth in the past 2 months.
2. Have a history of mental illness.
3. Severe liver and kidney dysfunction.
4. Cardiovascular, endocrine, hematopoietic system, immune system, and other primary diseases.
5. Drug-induced acne and occupational acne.
6. Patients who are unwilling to cooperate with acupuncture treatment.
7. In the course of treatment, other acne treatments are combined, such as oral or topical treatment with other drugs, laser therapy, and traditional Chinese medicine masks.

#### 2.2.5 Recruitment

Seventy six patients will be recruited through hospital outpatient clinics, advertisements on the hospital's social media (WeChat), and leaflets displayed by community service centers. The recruitment will provide a phone number so that potential patients can contact the researchers. The dermatologist will screen patients who meet the inclusion and exclusion criteria and use a digital camera (Canon EOS 200D) to take facial photos. The study assistant will be responsible for providing study information, such as the objectives, interventions, duration, benefits, and risks. Agreed to participate in the study patients will sign informed consent. The confidentiality of information recorded by patients will be protected.

#### 2.2.6 Randomization and allocation concealment

Patients will be randomly assigned to receive SSGA or conventional acupuncture at a ratio of 1:1. Sealed opaque envelopes will be applied to confirm random hiding. The random sequence number and group assignment information will be sealed in orderly envelopes. With the addition of the patients, these envelopes will be unfolded one by one in order, and the research assistant will record the assigned serial number in the case report form (CRF). The research assistant who does not participate in treatment or evaluation will keep these envelopes.

#### 2.2.7 Blinding

Patients, outcome assessors, and statisticians will turn a blind eye to this assignment. However, due to the characteristics of acupuncture, acupuncturists will not be deceived in this trial. Patients will not know their group status and patients in different treatment groups will be treated in different treatment rooms. Besides, they will be separated by a curtain to avoid communication during the trial. For the successful implementation of blinding, all researchers will be trained before the start of the trial.

### 2.3 Intervention

All patients will receive treatment for 8 weeks, twice a week (every Monday and Thursday), 30 minutes each time, a total of 16 sessions. Acupuncturists will be required to hold a Chinese medicine practitioner license and have more than 1 year of clinical experience. The treatment will use sterile disposable steel needles (Jiajian brand in Wuxi, Jiangsu, China: Suxie Zhuzhun 20152200225). According to the different acupuncture points and the depth of acupuncture, sterile disposable steel needles of different specifications will be selected. And acupuncture will be artificially stimulated for 10 seconds to achieve “Deqi”. That is, the subject will have special sensations such as soreness, numbness, swelling, and weight. And the surgeon has feelings of tightness under the needle.

#### 2.3.1 Group A

**Figure 2:**
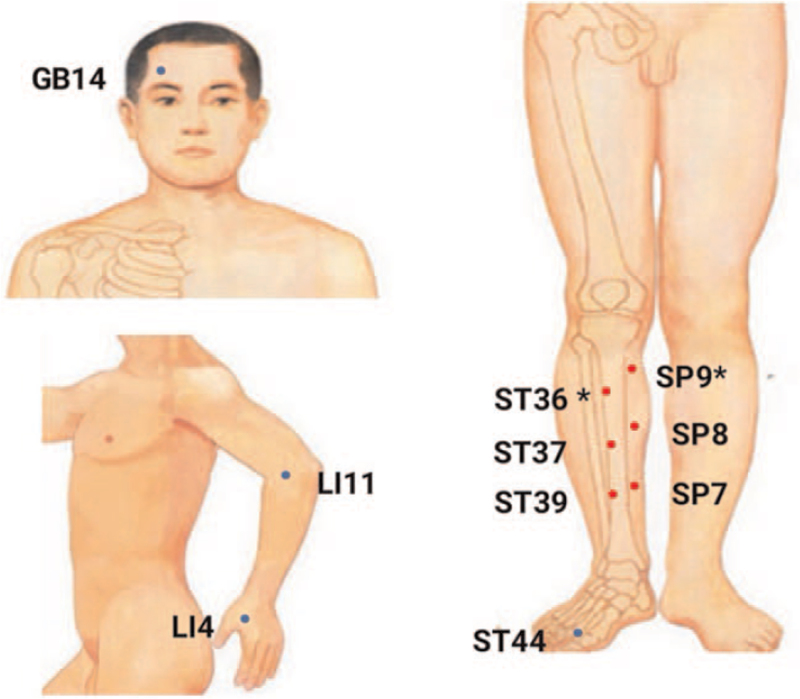
Locations of acupoints in the trial. The acupoints include SP9 Yinlingquan, SP8 Diji, SP7 Lougu, ST36 Zusanli, ST37 Shangjuxu, ST39 Xiajuxu, LI11 Quchi, LI4 Hegu, ST44 Neiting, GB14 Yangbai.

Group A will receive SSGA. The acupuncturist will use sterile disposable steel needles to stimulate left SP9 (Yinlingquan), left SP8 (Diji), left SP7 (Lougu), right ST36 (Zusanli), right ST37 (Shangjuxu), right ST39 (Xiajuxu). After routine disinfection, the acupuncturist will insert disposable steel needles vertically into SP9, SP8, SP7, ST36, ST37, and ST39. The specific selection of acupuncture points, needle specifications and the depth of acupuncture are shown in Table 1 and Figure 2 below.

**Table 1:** Locations for group A.

Acupoint	Location	Needle specifications	Depth of acupuncture
SP9 (Yinlingquan)	Medial condyle of the tibia and the medial border of the tibia	0.25 × 50 mm	25–40 mm
SP8 (Diji)	3 cun directly below SP9, posterior border of medial margin of tibia	0.25 × 50 mm	25–40 mm
SP7 (Lougu)	6 cun above the tip of the medial malleolus, posterior margin of the medial edge of the tibia	0.25 × 50 mm	25–40 mm
ST36 (Zusanli)	3 cun directly below ST35, and one finger-breadth lateral to the anterior border of the tibia	0.25 × 50 mm	25–40 mm
ST37 (Shangjuxu)	6 cun directly below ST35, and one finger-breadth lateral to the anterior border of the tibia	0.25 × 50 mm	25–40 mm
ST39 (Xiajuxu)	9 cun directly below ST35, and one finger-breadth lateral to the anterior border of the tibia	0.25 × 50 mm	25–40 mm

^a^1 cun (≈ 20 mm) is defined as the width of the interphalangeal joint of the patient's thumb.

#### 2.3.2 Group B

Group B will receive conventional acupuncture treatment. The acupuncturist will use sterile disposable steel needles to stimulate, bilateral LI11 (Quchi), bilateral LI4 (Hegu), bilateral ST44 (Neiting), bilateral GB14 (Yangbai), bilateral SP9 (Yinlingquan), bilateral ST36 (Zusanli). The acupuncturist will insert the disposable steel needles vertically into LI11, LI4, ST44, GB14, SP9, and ST36 after routine disinfection. The specific selection of acupuncture points, needle specifications, and the depth of acupuncture are shown in Table 2 and Figure 2 below.

**Table 2:** Locations for group B.

Acupoint	Location	Needle specifications	Depth of acupuncture
LI11 (Quchi)	The midpoint of the line between LU5 and lateral epicondyle of humerus	0.25 × 40 mm	20–30 mm
LI4 (Hegu)	Radial midpoint of the second metacarpal	0.25 × 25 mm	10–20 mm
ST44 (Neiting)	Between the second and third toes, the red and white flesh behind the webbed margin of the toe	0.25 × 25 mm	10–20 mm
GB14 (Yangbai)	1 cun on the eyebrow, pupils straight up	0.25 × 25 mm	7–15 mm
SP9 (Yinlingquan)	Medial condyle of the tibia and the medial border of the tibia	0.25 × 40 mm	20–30 mm
ST36 (Zusanli)	3 cun directly below ST35, and one finger-breadth lateral to the anterior border of the tibia	0.25 × 40 mm	20–30 mm

^a^1 cun (≈ 20 mm) is defined as the width of the interphalangeal joint of the patient's thumb.

### 2.4 Outcome measures

#### 2.4.1 Primary outcome

The primary outcome measurement will be expressed by the change in IGA score from baseline to the end of 8-week treatment. The IGA score is an overall assessment of the degree of inflammatory and non-inflammatory lesions and is divided into 6 levels.^[21,22]^

Grade 0 (0 points): the skin is smooth and clean, without any inflammatory or non-inflammatory lesions; Grade 1 (1 point): the skin is almost smooth, non-inflammatory lesions are rare, and no more than 1 small inflammatory lesion; Grade 2 (2 points): mild, with some non-inflammatory lesions, and only a few inflammatory lesions (only papules or pustules, no nodular lesions); Grade 3 (3 points): moderate, there are multiple non-inflammatory lesions, and there may be some inflammatory lesions, but no more than 1 small nodular lesion; Grade 4 (4 points): severe, with multiple non-inflammatory and inflammatory lesions, but only a few nodular lesions; Grade 5 (5 points): extremely severe, with multiple non-inflammatory and inflammatory lesions, and multiple nodular cystic lesions.

#### 2.4.2 Secondary outcome measurements

The secondary measurements will be measured by changes in IGA, total facial lesion counts (Total Lesion Counts),^[21,23]^ inflammatory lesion counts, non-inflammatory lesion counts,^[21]^ and Acne-Specific Quality of Life^[24]^ at 8 and 12 weeks compared with baseline display.

Total lesion count includes inflammatory lesion counts and non-inflammatory lesion counts. Inflammatory lesions include inflammatory papules, pustules, cysts, and nodules. Non-inflammatory lesions include blackheads and whiteheads. Lesions will be evaluated on the forehead, cheeks, nose, and chin.

Acne-specific quality of life has 14 items in total, covering 3 areas, including self-perception, social function, and emotional function.^[24]^ This scale is mainly used to test the quality of life of patients with skin diseases. Each item is divided into 4 levels according to the specific conditions of the patient, with a score of 0 to 3. The total score of 16 items is the patient's skin disease quality of life score. The total index score is 30 points. The higher the score, the worse the patient's quality of life.

The Expectation and Credibility of treatment rating scale will be used to measure the patients’ attitudes to acupuncture after the first treatment. The patients will be asked the following question: “Do you think SSGA will be effective in treating the symptoms of ance?”

#### 2.4.3 Data collection and management

The data for this study will be collected by the assistant and filled in CPFs. The independent investigator will enter the relevant information and data of this study into an Excel spreadsheet to protect its confidentiality. No one outside the research group will have access to relevant information. Participants’ research information may not be published outside of the research if there is no written permission from the participants.

### 2.5 Statistical analysis

#### 2.5.1 Clinical data analysis

Statistical analysis of all data will use IBM SPSS statistical version 24.0 software (IBM Corporation, Armonk, New York). For continuous variables, the data will be represented by the mean (standard deviation) or median (interquartile range) according to the normal distribution. Enumerated data will be displayed in percentage. For the primary outcome, we will calculate the IGA score at 8 weeks, and use the χ^2^ test to compare group A and group B. For secondary outcomes, continuous variables including IGA, TCL, Inflammatory lesion count, Non-inflammatory lesion count, Qol-Acne, and the Expectation and Credibility of acupuncture treatment will be compared at 8 and 12 weeks, using an unpaired Student *t* test or Wilcoxon rank-sum test (depending on the situation). A *P* value of <.05 will be considered a limit of statistical significance.

#### 2.5.2 Sample size

The total sample size was 76 cases and they are randomly divided into 2 groups. According to the trial design, the sample size estimation method used a completely random design sample rate and the population rate comparison. The sample size is calculated according to the following formula: $\text{n}=\pi_{0}\left(1-\pi_{0}\right){\left\{\frac{{\text{z}}_{\alpha }+{\text{z}}_{\beta }}{\delta }\right\}}^{2}$. Through literature study on ordinary acupuncture treatment for acne patients, the average effective rate was 76.7%, so the overall rate of Group A was *π*_0_ = 76.7%. Combined with clinical experience, the total effective rate of SSGA in treating acne is 98.48%, then the overall rate of expected test results was *π* = 98.48%. One-sided cutoff of this trial: z_α_ = 1.645, z_β_ = 1.282, δ=*π*-*π*_0_ = 0.9848-0.767 = 0.2178. Enter the formula to get the estimated sample content value n≈32. Considering that “dropping” may increase the sample size by up to 20%, the sample size was estimated to be 38 cases. Finally, Group A and Group B are according to the random number table method.

### 2.6 Quality control and safety monitoring

To promote patient retention and complete follow-up, total interventions to patients are free of charge. The data relating to this study will be recorded in the CRFs, and then input into the Excel spreadsheet by independent investigators as the first level of control to guarantee the accuracy of the data. The second level of data integrity will include data integrity validation and data monitoring throughout the study carried out regularly. All original forms (including the consent forms and CRFs) will be safely stored in Dongzhimen Hospital of Beijing University of Chinese Medicine. The Research Ethics Committee of Dongzhimen Hospital of Beijing University of Chinese Medicine will audit the trial independently of the investigators every 5 months and will decide whether to end the study early.

**Figure 3:**
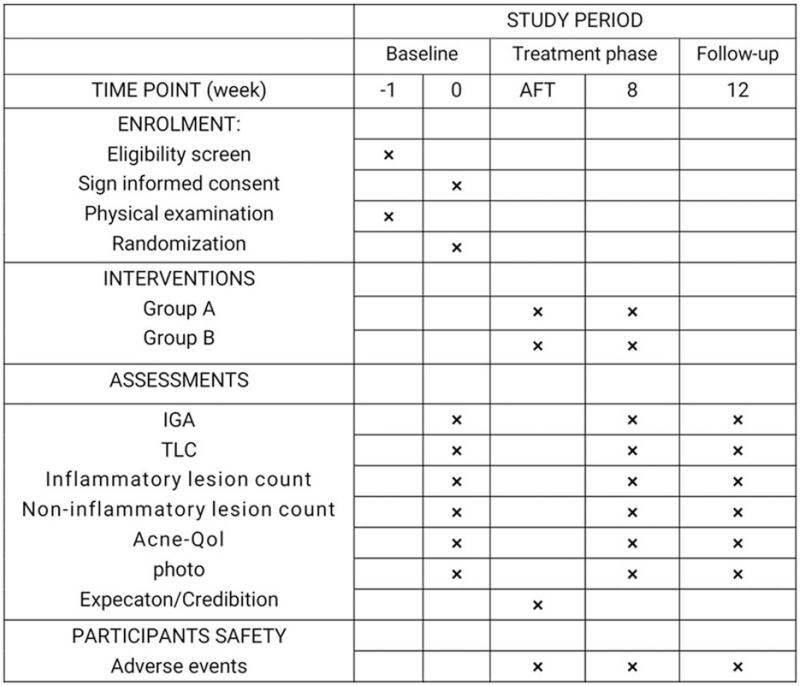
Template of content for the schedule of enrolment, interventions, and assessments. AFT = after the first treatment, TLC = total lesion count, IGA = investigation global assessment, Acne-Qol = acne-specific quality of life questionaire.

From baseline to week 12, adverse events and their numbers will be recorded. These adverse events will include adverse events related to acupuncture, such as needle fainting, infection, hematoma, sensation after acupuncture, as well as adverse reactions that cannot be related to acupuncture. For the safety assessment, the trial assistant will record the details of all adverse events in the CRF. Any serious adverse event will be reported to the ethical committees of Dongzhimen Hospital of Beijing University of Chinese Medicine within 24 hours, and the Dermatology Department will compensate for any injuries related to the intervention during the study period. The specific trial intervention process is shown in Figure 3 below.

## 3 Discussion

Acne is a chronic inflammatory skin disease, and its pathogenesis is still not completely clear. Modern studies suggest that its onset is related to the relative increase of androgens, increased secretion of sebum, hair follicles mouth of excessive and Propionibacterium acnes infection and so on. In addition, the immune imbalance, the role of inflammatory factors, and genetic related factors have also been recognized.^[11]^ The general effects of acne are physical sensory symptoms such as skin itching and pain. However, the most serious impact is on the quality of life. This influence is affected by various factors, which makes it difficult to quantitatively analyze. In youth, the self-confidence and self-esteem of acne sufferers are affected. If it can’t be cured, it may bring continuous negative psychological effects on adults, such as shame, embarrassment, depression, anxiety, and even suicide. Therefore, complementary and alternative medicine in modern practice regarding various dermatology is becoming more and more extensive.

As a unique alternative treatment method, acupuncture has the function of “external treatment of internal diseases” and can treat systemic diseases through the conduction of meridians and acupoints.^[25]^ It is widely used in China and has achieved good results in the treatment of acne, chloasma, eczema, allergic skin diseases, and other skin diseases.^[13]^ However, the existing trials only provide routine treatment for this disease and do not conduct syndrome differentiation.^[26–30]^ TCM treats acne and adopts the principle of syndrome differentiation and treatment. Starting from the origin, the advantages are significant.^[31,32]^ Therefore, under the guidance of TCM theory and clinical experience, we have designed a study on acne with the gastrointestinal damp-heat syndrome. This trial is based on the guidance of TCM theory. We aim to evaluate the changes in the count of facial skin lesions and the improvement of quality of life in patients with acne by SSGA. This study is a multi-center clinical randomized trial, which is more in line with clinical practice and closer to the real world. Moreover, the selection of acupoints is within a relatively safe range of the lower limbs, avoiding operations on the local lesions, so the patients’ acceptance is relatively high.

### 3.1 Limitation

Some limitations of our trial should be recognized. First of all, this trial is only for a certain type of acne, which is relatively limited. But this type of acne is the most common in the clinic, therefore we also cure and observe the majority of acne patients. Second, as a study, the sample size of this trial is relatively small. We will conduct a larger sample size study based on this trial to verify the effectiveness and safety of SSGA again in the future. Last, although this study cannot blind the acupuncturists, the patients will be blind to the group assignment well. Both treatments for the two groups of patients are real acupuncture, but the selected acupoints are different. These patients in both groups will have the feeling of “Deqi” during acupuncture. That is, they will have special sensations such as acidity, numbness, sinking, and swelling. In general, we designed this study and hope that this trial will provide favorable evidence for the treatment of gastrointestinal damp-heat by SSGA.

## Acknowledgments

We would like to thank all participants who contributed to this research.

## Author contributions

**Data curation:** Hui-Shang Feng, Guang-An Wang.

**Funding acquisition:** Chao-Qun Yan, Kai-Bing Tian.

**Investigation:** Hui-Shang Feng, Ya-Qi Yao.

**Literature retrieval:** Ping-Ping Duan, Chao-Qun Yan.

**Recruiting patients:** Ping-Ping Duan, Hui-Shang Feng, Ning Sun, and Ya-Qi Yao.

**Software:** Ping-Ping Duan, Yuan Chen, Kai-Bing Tian.

**Supervision:** Guang-An Wang.

**Writing – original draft:** Ping-Ping Duan, Chao-Qun Yan, Hui-Shang Feng, Ning Sun.

**Writing – review & editing:** Ping-Ping Duan, Guang-An Wang.
